# Prevalence and serotypes of *Salmonella* spp. on chickens sold at retail outlets in Trinidad

**DOI:** 10.1371/journal.pone.0202108

**Published:** 2018-08-23

**Authors:** Anisa S. Khan, Karla Georges, Saed Rahaman, Woubit Abdela, Abiodun A. Adesiyun

**Affiliations:** 1 School of Veterinary Medicine, Faculty of Medical Sciences, University of the West Indies, St. Augustine, Trinidad and Tobago; 2 Veterinary Public Health Unit, Ministry of Health, Port of Spain, Trinidad and Tobago; 3 Department of Pathobiology, College of Veterinary Medicine, Nursing and Allied Health, Tuskegee University, Tuskegee, United States of America; University of Campinas, BRAZIL

## Abstract

**Background:**

This cross-sectional study determined the prevalence of *Salmonella* spp. and their serotypes on dressed chicken sold at retail outlets in Trinidad. The study also investigated the risk factors for contamination of dressed carcasses by *Salmonella* spp. at cottage poultry processor outlets where chickens are slaughtered and processed for sale.

**Methods:**

A total of 133 dressed, whole chickens and 87 chicken parts from 44 cottage poultry processors and 36 dressed, whole chickens and 194 chicken parts from 46 supermarket outlets were randomly collected throughout the country. Isolation and identification of *Salmonella* spp. were performed using standard bacteriological techniques. Serotyping was performed by a regional reference laboratory.

**Results:**

The prevalence of *Salmonella* spp. in chicken carcasses sampled from cottage poultry processors and supermarkets was 20.5% and 8.3% respectively (p <0.001). The frequency of isolation of *Salmonella* spp. at cottage poultry processors was 22.4%, 23.0%, 7.1%, and 10.0% for non-chilled whole chicken, non-chilled chicken parts, chilled whole chicken and chilled chicken parts respectively. Fresh, non-chilled chicken (22.6%) yielded a higher frequency of isolation of *Salmonella* spp. than chilled chickens (8.3%). For supermarket samples, the frequency of isolation of *Salmonella* spp. was 19.0%, 8.1%, 0.0% and 7.6% for chilled whole chickens, chill chicken parts, frozen whole chicken and frozen chicken parts respectively. The swab method of sampling yielded a statistically significantly (p = 0.029) higher frequency (3.2%) of *Salmonella* spp. than the rinse method (1.6%). The predominant serotypes isolated were Kentucky (30.9%) and Javiana (22.7%). Use of chilled water-bath to cool carcasses was the only risk factor significantly (p = 0.044) associated with isolation of *Salmonella* spp.

**Conclusion:**

Raw chicken carcasses purchased from cottage poultry processors pose a significantly higher risk of contamination with *Salmonella* spp. than those sold at supermarkets.

## Introduction

Globally, despite the institution of several control measures, *Salmonella* infections continue to be problematic with millions of cases occurring annually, both in humans and animals [1]. The annual incidence of human salmonellosis in the world has been estimated to be 93.8 million cases [2].

Poultry products have particularly served as vehicles of *Salmonella* spp. which have caused human and animal diseases as well as economic losses. Worldwide, foodborne infections are under-reported but the problem is exacerbated in developing countries where infections and diseases are often grossly under-reported making it difficult to assess the magnitude of the problem [3]. However, it has been documented that the occurrence of chicken-borne salmonellosis in European Union member states and the United States of America can vary from 15.7% [4] to 85.0% [5] in humans, respectively.

The under-reporting of salmonellosis is of concern particularly in developing countries where diagnostic facilities and qualified personnel required to conduct investigations are inadequate [3, 6]. This has posed challenges in tracing the sources and causative agents implicated in epidemics.

In developing countries, the wet markets or cottage poultry processors serve as important sources of chicken consumed by the population. At these outlets, prevalence rates for *Salmonella* spp. in raw dressed chicken can vary among countries and between outlets as documented in Korea, 42.7% [7], Vietnam, 45.9% [8], India, 65.0% [9], and Malaysia, 100.0% [10]. Poor sanitary practices which are known to be prevalent at these often unregulated outlets that are not monitored by the appropriate authorities have contributed to a high frequency of isolation of *Salmonella* spp. from chicken carcasses [10,11].

Many risk factors have been associated with the isolation of *Salmonella* spp. at the retail outlets, particularly at the wet markets or cottage poultry processors. These include the size of outlets (wet markets, supermarkets and independent poultry stores) [8,11,12], sources of chickens (integrated and non-integrated systems) [12], storage temperature (ambient, chilling and freezing) [8,12], region of the country [13] and chicken rearing types (conventional and organic) [14], amongst other factors.

Commercial outlets, such as supermarkets, also serve as a source of chicken contaminated by *Salmonella* spp. Reported prevalence of *Salmonella* spp. have ranged from 4.0% to 20.0% in developed countries [15,16,17] where stringent measures have been instituted to control salmonellosis on poultry farms and good sanitary practices at poultry processing plants and sale outlets [18,19] compared with 43.0% to 62.5% in developing countries, where such sanitary practices are uncommon [8,20,21,22].

Reported prevalence for *Salmonella* spp. vary globally but these rates are affected by the isolation procedures which have different sensitivity and specificity [23]. Conventional methods have varied both in sample type, i.e. rinse method [24,25,26,27], whole carcass enrichment [28,29,30] and swab method [10,31], and in the use of non-selective enrichment media [32,33] and selective enrichment media [34].

The serotypes of *Salmonella* spp. isolated from chicken carcasses sampled from retail outlets have varied by country [35,36] but some serotypes such as *S*. Enteritidis, *S*. Typhimurium, *S*. Infantis, *S*. Newport, and *S*. Derby have repeatedly been recovered from chickens and associated with poultry-borne infections or outbreaks in humans [6,15,18]. Some reports have also documented the changes in the predominant serovars, for example from *S*. Enteritidis to *S*. Heidelberg and *S*. Kentucky in the USA [37] and *S*. Enteritidis to *S*. Derby in Uruguay [38], respectively.

Since 1989, *S*. Enteritidis has emerged as pathogen of public health concern in the Caribbean region, causing sporadic cases and outbreaks of diarrhea in both the local and tourist populations [6]. There is a dearth of information on the risk of salmonellosis posed by chicken sold at cottage chicken processors as well as chicken carcasses sold by supermarkets. A study conducted in 2006 [32] reported a prevalence of 7.3% for *Salmonella* spp. from chicken at selected cottage processing outlets and another study reported a prevalence of 71.4% for *Salmonella* spp. in chicken from cottage processors in one region of the country [39]. However, both studies did not evaluate the risk factors for *Salmonella* contamination at these outlets. In 2012, Dookeran et al. [40] observed that broiler carcass contamination with *Salmonella* spp. increased during transportation from the farm to the processing plant, during processing and at retail outlets. Moreover, there are no data on the prevalence of *Salmonella* contamination of chicken meat sold by supermarkets in the country.

In Trinidad and Tobago, cottage poultry processors and supermarkets have been reported to be the source of 55% and 45% respectively of the chicken consumed in the country [39]. To date, there is a lack of current information on the prevalence and characteristics of *Salmonella* spp. from both types of retail outlets (cottage poultry processors and supermarkets). Therefore, the objectives of this study are to determine the prevalence and serotypes of *Salmonella* spp. in dressed broiler meat sold at retail outlets in Trinidad. The study also assesses the risk factors for *Salmonella* contamination of broiler carcasses at the cottage processing outlets.

## Methods

### Consent of the owners of cottage processor outlets and Ethics Committee approval

The consent of the owners of the Cottage Processor outlets were obtained before they completed the questionnaires and chickens were purchased for the study. In addition, the Ethics Committee of the Faculty of Medical Sciences, University of the West Indies, St. Augustine, approved the protocol before the study commenced.

### Retail outlets for broiler chickens in Trinidad and Tobago

The retail outlets included in this study comprised primarily the cottage poultry processors also known as ‘pluck shops’ and supermarkets.

Cottage poultry processors are roadside establishments that slaughter and process poultry for customers on request, providing fresh whole chickens or chicken parts (cut-up whole chickens). Most of these outlets offer for sale recently slaughtered chickens while a few sell chilled chickens. At the supermarkets, only chilled and frozen chicken carcasses or parts are sold.

The ‘pluck shops’ are conveniently located throughout each county across the country. The operations at these outlets comprise a pen or area where birds are kept prior to slaughter, plastic or galvanize cones that hold the birds prior to and after severing of the jugular vein, large pots/vessels with hot water for scalding birds prior to defeathering, defeathering machine/drums, counter-tops where carcasses are eviscerated and sink/container where carcasses are rinsed with tap water. Some establishments utilize a chilled/iced water bath to cool carcasses before they packaged for sale. Practices at these outlets are often not monitored by the designated authority due to manpower shortage.

Supermarkets are outlets of various sizes that retail poultry meat along with other products. Chicken carcasses originate from local commercial chicken processing plants, where meat inspectors from the Veterinary Public Health Unit conduct routine inspection. Some supermarkets also retail chicken carcasses from other countries.

### Location of broiler chicken retail outlets

Retail outlets were located and identified from the information provided by the Poultry Surveillance unit, Veterinary Public Health Unit and Association of Supermarket owners. Overall, a total of 265 cottage poultry processor outlets and 125 supermarkets and their locations were identified.

### Sources and number of samples studied

The number of samples collected for the study was estimated using the formula for estimating prevalence for an infinite population size [41] and the estimated minimum sample size was determined to be 384. However, a total of 450 samples were collected. They were represented by 220 samples from outlets of cottage poultry processors and 230 samples from supermarkets. The sampling unit at each retail outlet was five dressed chickens, comprising whole carcasses and chicken parts.

### Selection of retail outlets for the study

The cottage processor outlets were selected based on proportional sampling from each of the seven counties in Trinidad using simple random sampling method. Overall, a total of 44 cottage processing outlets were selected. Supermarkets were categorized into sizes based on the number of cashiers at each establishment and the selection from each group were done using proportional sampling and simple random sampling method within each group of supermarkets.

For the study, 46 supermarkets comprising 18, 14, 9 and 5 outlets classified as chain supermarkets (more than 1 outlet), large supermarkets (4 or more cash registers), medium supermarkets (2–3 cash registers) and small supermarkets (1 cash register only) were included in the sampling plan.

### Administration of questionnaire

At each selected cottage processor outlet, consenting owners, after being briefed about the project, were administered a questionnaire to elicit information on demographic data and risk factors for carcass contamination with *Salmonella* spp. These included the number of workers employed, levels of workers’ training, experience of workers, source of live birds, sale activity, sanitary practices (scored by a sanitation score sheet: S1 Appendix), and presentation of the carcasses at outlets. The detailed questionnaire is available in S2 Appendix. At supermarkets, information obtained included the source of chickens (processing plants) and presentation (chilled or frozen) for sale.

### Processing of samples

At each retail outlet, the five chicken carcasses were collected in separate sterile bags and transported on ice to the laboratory for processing within 3 h. In the laboratory, the initial processing of the carcasses or carcass parts followed the procedure described by Rodrigo et al. [32]. Subsequently the chicken samples were each processed using both the carcass swabs and carcass rinse water.

#### Swab method

A slight modification of earlier reported carcass swab-sampling method [42] was used. Internal and external swabs of each carcass were taken using sterile cotton swabs and placed in 9 ml Buffered Peptone Water (BPW) (Oxoid, Hampshire, England).

#### Rinse method

The same carcasses that were swabbed were then processed by a carcass rinse method [26,32], a process different from the whole carcass enrichment method [29]. Six milliliters of Phosphate Buffered Saline (PBS, pH 7.2) per gram of carcass weight was placed in a sterile bag and the carcass was massaged and rotated no less than 25 times. From the resulting rinsate, 25 ml were aseptically collected and centrifuged at 4470 g for 20 minutes after which 1 ml of sediment was removed and transferred to 9 ml BPW.

### Non-selective and selective enrichments of samples

Both pre- enriched BPW samples (swab and rinse method) were incubated at 37°C for 18–24 h. The samples in enrichment media were then selectively enriched in tetrathionate (TT) broth (Oxoid, Hampshire, England) and Rappaport-Vassiliadis Soya (RVS) broth (Oxoid, Hampshire, England) and incubated for 18–24 h at 37 and 42°C respectively.

### Isolation and identification of *Salmonella* serotypes

Samples enriched in the selective media (TT and RVS) were sub-cultured onto xylose lysine tergitol 4 (XLT-4; Oxoid, Hampshire, England) and brilliant green agar (BGA; Oxoid) and incubated aerobically at 37°C for 18–24 h. Suspected *Salmonella* colonies (pink isolated colonies on BGA, red colonies with black centers on XLT-4) were subjected to biochemical tests for identification of *Salmonella* spp. using standard methods [43]. All isolates biochemically confirmed to be *Salmonella* spp. were subjected to serological typing using *Salmonella* polyvalent antiserum (A-I and Vi, Difco, Detroit, MI). Complete confirmation and serotyping of *Salmonella* isolates representative of those recovered by the carcass rinse/swab, RVS/TT and BGA/XLT-4 methods were done at the Public Health Laboratory, Ministry of Health, Barbados.

### Statistical analyses

Chi-square analyses were conducted using the Statistical Package for Social Sciences, SPSS (version 23, IBM Corp., Somers, NY) to determine whether there were statistically significant differences in the frequency of isolation of *Salmonella* spp. amongst (i) risk factors associated with *Salmonella* contamination, (ii) types of outlets (cottage poultry processors/supermarkets), (iii) carcass presentation (whole chicken/chicken parts), (iv) temperature at point of sale (ambient/chilling/freezing temperatures), and (v) method of bacterial isolation (carcass swab/carcass rinse).

The level of significance was determined at an alpha level of 0.05. Additionally, a mixed effects logistic regression model was constructed with the presence of *Salmonella* in carcasses sampled as the outcome variable and supermarket size (large *versus* others) and whether it was a chain or not, as covariates. The supermarket identification was set a random variable to determine the degree of clustering. For cottage processors, county was used as covariates and cottage processor identification was set as a random variable. The intra-cluster correlation coefficient (ICC) was calculated to measure the proportion of variation between groups as a measure of the total between and with group variation. Data was analysed using STATA 12 (STATA Corporation, College Station Texas).

## Results and discussion

The relationship of the frequency of isolation of *Salmonella* and eight risk factors studied at the outlets of cottage operators are shown in Table 1. The only factor that had statistically significant association with isolation of *Salmonella* spp. was the use of chilled water-baths to cool carcasses post-slaughter. In this study, carcasses purchased from outlets that used chilled water had significantly higher prevalence of *Salmonella* spp. (66.7%) compared to outlets that did not (25.7%). It is significant that the chilling water-bath often contained stagnant water, which if not frequently changed, may result in a buildup of contamination and cross-contamination of carcasses with *Salmonella* spp. and other pathogens. This agrees with published results particularly in road-side poultry processing outlets as reported by others [11, 20, 44, 45]. Infrequent change of chilling water may also lead to an increase of the temperature of the chilling water tank as warm carcasses are constantly added. It has been reported that high temperature of the water bath for chilling may also lead to multiplication of bacteria [46].

**Table 1:** Risk factors for contamination of chicken carcasses by *Salmonella* spp. at outlets of cottage poultry processors in Trinidad.

Risk Factor	No. of cottage poultry processor outlets sampled	No. (%) positive for *Salmonella* spp.	P-value
**Sale activity**^a^**: Weekday**			1
Medium	25	9 (36.0)	
Large	18	6 (33.3)	
**Sale activity: Weekends**			1
Medium	24	8 (33.0)	
Large	20	7 (35.0)	
**Source of live birds**			0.801
Source A	11	2 (18.2)	
Source B	21	6 (28.6)	
Source C	10	4 (40.0)	
Source D	21	7 (33.3)	
Source Others	13	5 (38.5)	
**Frequency of changing of rinse water**			1
Every 20 birds or less	27	9 (33.3)	
Every 21 birds and greater	17	6 (35.3)	
**Frequency of general cleaning of work area**			1
Once daily	32	11 (34.4)	
More than once daily	12	4 (33.3)	
**Frequency of thorough cleaning of work area**			1
At least once a week	29	10 (34.5)	
Less than once a week	15	5 (33.3)	
**Frequency of cleaning pens**			0.761
At least once every 3 weeks	15	5 (33.3)	
Every 1–6 months	13	6 (46.2)	
**Use of chilled water bath**			0.044
Yes	9	6 (66.7)	
No	35	9 (25.7)	
**Chicken sale from counter**			0.976
Yes	16	6 (37.5)	
No	28	9 (32.1)	

^a^ Medium; <350 birds; Large, 351 or more birds.

Although the frequency of isolation of *Salmonella* spp. across broiler farm sources ranged from 18.2% (Source A) to 40.0% (Source C), the differences were however not statistically significant (P>0.05; X^2^). This study is not in agreement with Donado et al. [47] who reported that the source (integrated *versus* non-integrated) of live chickens from retail outlets in Columbia significantly affected the frequency of isolation of *Salmonella* spp. from chicken carcasses. This may be explained, in part, by the apparent low prevalence of *Salmonella* spp. in live chickens from broiler farms in the country, ranging from 0.0% to 5.0% [48]. In our study the practices observed at the outlets resulting in cross-contamination, due to the use of chilled water-baths, may be more important contributors to carcass contamination than sources of live chickens. Nidaullah et al. [10] reported a high prevalence of *Salmonella* spp. in carcasses due to cross contamination throughout the various stages ofprocessing at wet markets. Rivera-Perez et al. [49] also investigated the risk points during broiler carcass processing and reported that *Salmonella* contamination increased from 10% to 40% during evisceration and subsequent spray washing.

Of a total of 44 cottage processor outlets sampled across the seven counties, the frequency of isolation of *Salmonella* spp. was 34.1% (15/44). In each county (7/7; 100.0%) samples positive for *Salmonella* were found. (Table 2). The range of outlet positivity for *Salmonella* spp. was from 21.4% (Victoria) to 75.0% (Nariva/Mayaro) (p = 0.650). A study conducted in 2006 in the county of Mayaro [39] reported a prevalence of 71.4% for *Salmonella* spp. at broiler retail outlets, which agrees with our study.

**Table 2:** Frequency of isolation of *Salmonella* spp. from broilers sold at retail outlets across Trinidad.

**Type of retail outlet**	**County**	**Total No. of Cottage poultry processors**^a^	**No. (%) of Cottage poultry processors sampled**	**No. (%) of Cottage poultry processors positive for *Salmonella* spp.**^b^	**P-value**	**No. of chickens tested**	**No. (%) of chickens positive for *Salmonella* spp.**^b^	**P-value**
**Cottage poultry processors**	St George Central	17	3 (17.6)	1 (33.3)	0.65	15	5 (33.3)	<0.05
	St George East	27	5 (18.5)	2 (40.0)		25	4 (16.0)	
	St Andrew/St David	19	3 (15.8)	1 (33.3)		15	1 (6.7)	
	Nariva/Mayaro	23	4 (17.4)	3 (75.0)		20	11 (55.0)	
	Caroni	40	7 (17.5)	2 (28.6)		35	3 (8.6)	
	Victoria	91	14 (15.4)	3 (21.4)		70	10 (14.3)	
	St Patrick	48	8 (16.7)	3 (37.5)		40	11 (27.5)	
	Sub-Total	265	44 (16.6)	15 (34.1)		220	45 (20.5)	
**Supermarkets**	**Group of supermarkets**	**Total No. of supermarkets**^c^	**No. (%) of supermarkets sampled**	**No. (%) of supermarkets positive for *Salmonella* spp.**^b^	**P value**	**No. of chickens tested**	**No. (%) of chickens positive for *Salmonella* spp.**^b^	**P value**
	Chain	74	18 (24.3)	3 (16.7)	0.107	90	4 (4.4)	0.023
	Large	18	14 (77.8)	7 (50.0)		70	11 (15.7)	
	Medium	17	9 (52.9)	1 (11.1)		45	1 (2.2)	
	Small	16	5 (31.3)	2 (40.0)		25	3 (12.0)	
	Subtotal	125	46 (36.8)	13 (28.3)		230	19 (8.3)	
	Total	390	90 (23.1)	28 (31.1)		450	64 (14.2)	

^a^Total number of cottage poultry processors in Trinidad.

^b^Based on the use of both rinse and swab methods.

^c^Group of supermarkets: Chain- supermarket having more than one (1) outlet; Large- supermarket having 4 or more cash registers; Medium- supermarket having 2–3 cash registers; Small- supermarket having only 1 cash register.

At supermarkets, there was no difference in the odds of isolating *Salmonella* from large supermarkets *versus* other types of establishments or chain outlets. An ICC of 0.22 < 95% CI, 0.18–0.737> was calculated, which indicates that 22% of the variation in detecting *Salmonella* is due to between supermarket factors and 78% is explained by differences within supermarkets.

As for cottage chicken processors, there was no difference in the odds of detecting *Salmonella* according to county in which the cottage processor outlet was located. An ICC of 0.80 <95% CI, 0.63–0.91> was calculated which indicates that 80% of the variation in the detection of *Salmonella* in chickens sampled is explained between cottage chicken outlets and 20% is within samples from these cottage chicken outlets. This indicates a high level of clustering in the isolation of *Salmonella* at the level of the outlets.

Of a total of 220 chickens sampled from cottage poultry processors, 45 (20.5%) were positive for *Salmonella* spp. using both the carcass rinse and carcass swab methods. In a study conducted in 2006 using only the rinse method, Rodrigo et al. [32] reported a considerably higher prevalence of *Salmonella* spp. (7.3%) for broilers sampled from cottage poultry processors (‘pluck shops’) compared to a prevalence of only 2.3% (5/220) in the current study using a similar rinse method. Cox et al. [50] reported that there may be an increase or decrease in prevalence based on sampling technique and methods used.

In the current study, the frequency of isolation of *Salmonella* spp. from carcasses ranged from 6.7% (county St. Andrew/St. David) to 55.0% (county Nariva/Mayaro). The differences were statistically significant (P<0.05). However, different results were obtained by Rodrigo et al. [32] that reported a prevalence of 25.0% in county St. Andrew/St. David and 0.0% in counties Caroni and Victoria and 8.3% in county Nariva/Mayaro. The differences between the two studies could be due, in part, to a true difference in prevalence or differences in methodologies. There have been several reports of higher sensitivity for the isolation of *Salmonella* spp. with buffered peptone water (BPW), Rappaport Vassiliadis Soya broth (RVS) and xylose-lysine decarboxylate- 4 (XLT-4) [51,52,53] compared to the media used in the study by Rodrigo et al. [32]. The implication of our finding is that chicken purchased at cottage poultry processors in county Nariva/Mayaro pose a higher risk of exposure to *Salmonella* spp. to consumers if improperly cooked. The frequency of isolation of *Salmonella* spp. from broilers from cottage poultry processors in Trinidad and Tobago is lower than those reported for wet markets and similar outlets in Nepal, 38.0% [54], Vietnam, 48.9% [8], China, 52.2% [11], Bangalore- India, 65.0% [9] and Malaysia; 100% [10].

Of a total of 46 supermarkets visited, 13 samples (28.3%) yielded *Salmonella* spp. with a range of 11.1% (Medium-sized outlets) to 50% (Large-sized outlets) (p = 0.107). Nineteen (8.3%) of 230 chicken carcasses sampled were positive for *Salmonella* spp. (p = 0.023). Studies conducted at supermarket outlets elsewhere have documented higher prevalence of *Salmonella* spp. as in Vietnam, 43.0% [55] and in China, 51.2% [11] where large supermarkets accounted for 50.3% and small supermarkets, 52.1% of the positive samples, respectively. The differences may be accounted for by many factors including high prevalence of *Salmonella* spp. in live broilers at slaughter, hygienic practices during slaughter at the wet markets [10,55] and sensitivity of the laboratory techniques used in the isolation of the organism [56].

It is important for food safety to have detected that the prevalence (20.5%) of *Salmonella* spp. from cottage processor outlets was statistically significantly higher (P<0.05) than the prevalence found in supermarkets (8.3%). Therefore, chicken carcasses purchased from cottage poultry processors in Trinidad pose a higher risk of salmonellosis than those that originate from supermarkets. On the contrary, studies conducted by Yang et al. [11] in China and Ta et al. [8] in Vietnam reported no significant differences in the isolation rates from chickens sampled at wet markets and supermarkets. The reason for the higher isolation rate at cottage poultry processors could be due to cross contamination resulting from poor hygiene and manual handling of the chicken during processing thereby exacerbating cross contamination [10]. Additionally, the lower isolation rate from supermarkets in our study may be because most of the broiler chickens sold in supermarket outlets originated from commercial processing plants where sanitary practices are enforced by personnel of the Veterinary Public Health unit while no such quality control occurs at the cottage processor outlets.

Overall, 64 (14.2%) of 450 chicken samples from retail outlets (cottage poultry processors and supermarkets) were positive for *Salmonella* spp., a prevalence at the lower end of the 12–85% range for isolation of the pathogen from retail outlets worldwide [57].

The effects of the temperature (fresh/non-chilled, chilled and frozen) and type (whole and parts) of chicken carcasses presented for sale at the retail outlets on the frequency of isolation of *Salmonella* spp. are shown in Table 3. The range of isolation of *Salmonella* spp. based on temperature was from 0.0% for frozen chickens to 22.6% for fresh, non-chilled chickens (P>0.05). Of 190 fresh, non-chilled carcasses sampled, 43 (22.6%) were positive for *Salmonella* spp. whereas only 2 (8.3%) of 24 chilled carcasses sampled were contaminated by the pathogen (P>0.05). A possible reason for the lower isolation rate with a decrease in temperature could be because low temperatures have been reported to have bacteriostatic potential thereby causing injury to the bacterial cell wall, limiting its proliferation in non-selective and selective media [58]. In a review of 18 studies done worldwide, Huda et al. [57] reported that the highest contamination potential with *Salmonella* for chicken stored at room temperature was 37% with a lower and upper average of 24–29%. This is slightly higher than the 22.6% isolation rate detected in our un-chilled carcasses. Additionally, the authors reported that contamination of chicken stored at chilled and frozen temperatures were 34 and 35%, respectively. These findings are however higher than those detected in our study, where chilled and frozen chickens yielded *Salmonella* spp. at a rate of 8.3% and 0.0%, respectively. The differences in the duration of storage of chicken carcasses at ambient and chilling temperatures, which were not investigated in the current study, may also be responsible, in part, for our findings. Ta et al. [8] also failed to detect significant differences in the isolation rate of the organism with respect to storage temperatures (room temperature and chilled products), in agreement with our findings.

**Table 3:** Frequency of isolation of *Salmonella* spp. by presentation and temperature conditions at which chicken carcasses are kept at cottage poultry processors and supermarket outlets.

Non-chilled				Chilled			Frozen			All types	
Type of retail outlet	Type of chicken presentation	No. of samples tested	No. (%) positive for *Salmonella* spp.^a^	No. of samples tested	No. (%) positive for *Salmonella* spp.^a^	P- value	No. of samples tested	No. (%) positive for *Salmonella* spp.^a^	P-value	No. of samples tested	No. (%) positive for *Salmonella* spp.^a^
**Cottage poultry processors**	Whole	116	26 (22.4)	14	1 (7.1)	0.327	3	0 (0.0)	NA	133	27 (20.3)
	Parts	74	17 (23.0)	10	1 (10.0)	0.635	3	0 (0.0)		87	18 (20.7)
	Total	190	43 (22.6)	24	2 (8.3)	0.176	6	0 (0.0)		220	45 (20.5)
	P-value		1		0.804			NA			1
**Supermarkets**	Whole	NA	NA	21	4 (19.0)	NA	15	0 (0.0)	0.125	36	4 (11.1)
	Parts	NA	NA	37	3 (8.1)	NA	157	12 (7.6)	1	194	15 (7.7)
	Total	NA	NA	58	7 (12.1)	NA	172	12 (7.0)	0.097	230	19 (8.3)
	P-value	NA	NA		0.241			0.603			0.51

NA: Not applicable.

^a^Based on the use of both rinse and swab methods.

A comparison of the type of presentation (whole and part) of carcasses sampled from cottage processor outlets, revealed *Salmonella* isolation rates of 20.3% (27/133) and 20.7% (18/87) from whole carcasses and carcass parts, respectively (P>0.05). This suggests that the practice of cutting whole chickens into parts did not significantly increase *Salmonella* contamination.

For the samples collected from supermarket outlets where fresh, non-chilled chickens were not sold, the frequency of isolation of *Salmonella* spp. was 12.1% (7/58) for chilled carcass samples compared with 7.0% (12/172) for frozen carcasses but the difference was not statistically significant (p = 0.097). Considering the possible effect of temperature on types of presentation, for whole carcass samples the frequency of isolation from chilled chicken was 19.0% (4/21) compared to 0.0% (0/15) for frozen whole chicken but the difference was not statistically significant (p = 0.125). As for chicken parts, the frequency of isolation of *Salmonella* spp. was 8.1% (3/37) and 7.6% (12/157) for chilled chicken and frozen chicken respectively and the difference was not statistically significant (p = 1). *Salmonella* was isolated from 4 (11.1%) of the 36 whole carcasses and from 15 (7.7%) of the 194 frozen part samples but the difference was not statistically significant (p = 0.510). Since the minimum growth temperature for *Salmonella* on poultry meat is 5°C, with an optimum temperature ranging between 35–43°C [59], this could have contributed to the difference in isolation rates based on storage conditions. Morris and Wells [60] reported an increase in isolation rate from chilled carcasses rotated in an ice slush and suggested that extensive contact between carcasses may have contributed to cross-contamination. Additionally, Ahmed et al. [58] found that freezing below -18°C of *Salmonella*-positive carcasses eliminated its presence and suggested this was due to the low temperature damaging the bacterial cell wall leading to the death of the pathogen. Contrary to the previously mentioned studies and similar to our results, Yang et al. [11] found no significant difference between storage temperature and the isolation of *Salmonella* on raw poultry in China.

Of the 203 chicken samples of local origin collected from supermarket outlets, 18 (8.9%) were positive for *Salmonella* spp. while of the 27 samples of foreign origin, only 1 (3.7%) was positive (p = 0.707). The difference in frequency of isolation of *Salmonella* spp. from chicken obtained from local processing plants compared with those from plants in the USA sources, although not statistically significant, may reflect the fact that local chickens offered for sale at supermarkets originate from processing plants where thorough poultry inspection and sanitary practices are enforced, similar to that in the USA. The majority of imported poultry products to Trinidad originate from the USA.

The frequency of isolation of *Salmonella* spp. from chickens reared under the conventional production system was 8.1% (17/209), therefore similar (p = 0.687) to that observed for organically reared chickens (9.5%; 2/21). The low sample size of organically-reared chickens available for sampling in the current study was primarily because this is not a common practice in the country.

For chicken samples collected from supermarket outlets, the frequency of isolation of *Salmonella* spp. was similar for the rinse method (3.9%) and the swab method (3.5%) and the difference was not statistically significant (p = 1) as shown in Table 4. However, for chicken samples from cottage poultry processors, the swab method (9.5%) was more sensitive (p = 0.002) than the rinse method (2.3%). Overall, the swab method was determined to be a more sensitive method, 3.2% (29/450) than the rinse method, 1.6% (14/450, p = 0.029).

**Table 4:** Frequency of isolation of *Salmonella* spp. by the rinse and swab methods.

Source of sample	Types of sample processed	No. of samples tested	No. (%) positive for *Salmonella* spp. by:			
			Rinse method	Swab method	P-value	Total
**Supermarkets**	Whole chicken	58	2 (3.4)	5 (8.6)	0.438	7 (6.0)
	Parts	172	7 (4.1)	3 (1.7)	0.336	10 (2.9)
	Sub total	230	9 (3.9)	8 (3.5)	1	17 (3.7)
	P-value				0.057	
**Cottage poultry processors**	Whole chicken	133	3 (2.3)	15 (11.3)	0.007	18 (6.8)
	Parts	87	2 (2.3)	6 (6.9)	0.278	8 (4.6)
	Sub total	220	5 (2.3)	21 (9.5)	0.002	26 (5.9)
	P-value				0.195	
**Total**	Whole chicken	191	5 (2.6)	20 (10.5)	0.004	25 (6.5)
	Parts	259	9 (3.5)	9 (3.5)	1	18 (3.5)
	Sub total	450	14 (1.6)	29 (3.2)	0.029	43 (4.8)
	P-value				0.004	

In addition, the swab method yielded significantly a higher isolation rate for *Salmonella* spp. in whole chicken sampled at cottage poultry processors (11.3%; 15/133) *versus* the rinse method (2.3%; 3/133). Overall, for whole chicken samples (supermarkets and cottage poultry processors), 10.5% (20/191) were positive for *Salmonella* spp. by the swab method compared to 2.6% (5/191) by the rinse method (p = 0.004). The finding that there was no statistically significant difference in the isolation rate of *Salmonella* spp. by the two methods for the samples collected at the supermarkets, together with the lower frequency of isolation, may suggest differences in the quantitative level of contamination between meats sold at supermarkets and cottage poultry processors. Swab method is used for environmental sampling [10] and large animal studies where the entire carcass cannot be used for testing. Whilst the swab techniques are commonly reported to focus on one site, there have been reports where two or three sites and whole swab techniques were compared and it was found that multi-site swabbing yielded significantly higher isolation rates of bacteria compared with the swabbing of one site only [31, 61]. Additionally, whole carcass sampling via rinsing or swabbing was deemed necessary for optimum *Salmonella* recovery [31]. The findings in our study are however at variance with other studies which suggest that the swab method is not as sensitive as other methods such as excision of skin/muscle tissue or rinsing [42, 62].

The serotypes of *Salmonella* spp. isolated from the retail outlets were compared based on the type of samples collected (Table 5). Of a total of 68 isolates of *Salmonella* spp. from cottage processor outlets, 10 different serotypes were identified while 6 serotypes were identified among the 29 isolates from supermarket outlets. Whole carcasses accounted for 58.8% and 17.2% of the serotypes recovered from samples collected from the cottage poultry processors and supermarkets, and 38.2% and 82.8% for chicken parts respectively. The predominant serotypes detected at cottage poultry processors were *S*. Javiana (30.9%; 21/68), *S*. Kentucky; 23.5%; 16/68) and *S*. Manhattan (16.2%; 11/68) whereas at supermarkets *S*. Kentucky, *S*. subspecies enterica I and *S*. San Diego accounted for 47.3%, 27.6% and 10.3% of the serotypes, respectively.

**Table 5:** Serotypes of *Salmonella* spp. isolated from retail outlets and by type of sample.

Type of outlet	Serotype	No. (%) of isolates	Type of sample
**Cottage poultry processor**	Javiana	11 (16.2)	Whole carcass
	Kentucky	10 (14.7)	Whole carcass
	Manhattan	7 (10.3)	Whole carcass
	Warragul^a^	5 (7.4)	Whole carcass
	subspecies enterica I	2 (2.9)	Whole carcass
	Group D	2 (2.9)	Whole carcass
	Bloomsbury	2 (2.9)	Whole carcass
	Group C	1 (1.5)	Whole carcass
	Javiana	10 (14.7)	Chicken parts
	Kentucky	6 (8.8)	Chicken parts
	Manhattan	4 (5.9)	Chicken parts
	Aberdeen	3 (4.4)	Chicken parts
	Warragul^a^	2 (2.9)	Chicken parts
	Schwarzengrund	1 (1.5)	Chicken parts
	Subtotal	68 (70.1)^b^	Chicken parts
**Supermarket**	Kentucky	4 (13.8)	Whole carcass
	Javiana	1 (3.4)	Whole carcass
	Kentucky	10 (34.5)	Chicken parts
	subspecies enterica I	8 (27.6)	Chicken parts
	San Diego	3 (10.3)	Chicken parts
	Montevideo	2 (6.9)	Chicken parts
	Westhampton	1 (3.4)	Chicken parts
	Subtotal	29 (29.9)^c^	Chicken parts
	Total	97	

^a^ Possible *Salmonella* Warragul

^b^ Of a total of 68 isolates

^c^ Of a total of 29 isolates

At cottage poultry processors, serotypes *S*. subspecies enterica I, *S*. Group D, *S*. Bloomsbury and *S*. Group C were detected in whole carcasses but not in chicken parts, whereas serotypes *S*. Aberdeen and *S*. Schwarzengrund were detected in chicken parts and not in whole carcasses.

At supermarkets, the serotypes detected differed between the types of sample, with *S*. Javiana being detected in whole carcasses but not in chicken parts. Serotypes detected in chicken parts but not in whole carcasses included *S*. subspecies enterica I, *S*. San Diego, *S*. Montevideo and *S*. Westhampton. Overall, serotypes detected in whole carcasses alone included *S*. Group D, *S*. Bloomsbury and *S*. Group C while serotypes being detected in chicken parts alone comprised *S*. Aberdeen, *S*. Schwarzengrund, *S*. San Diego, *S*. Montevideo and *S*. Westhampton. The differences in the serotypes of *Salmonella* spp. between the different type of sample could have been due to differences in origin and source of broiler chickens, for example importation, which were not determined in the current study.

Previous studies conducted in the country and the Caribbean region on broilers, layers and eggs have detected several serotypes. Notably Rodrigo et al. [32] reported isolation of serotypes *S*. Kiambu, *S*. Kentucky and *S*. Mbandaka in broilers, whereas Adesiyun et al. [48] detected *S*. Typhimurium in Trinidad. In Jamaica, *S*. Austenborg and *S*. Kentucky were isolated from broilers [63]. Studies conducted on eggs and layer farms detected *S*. Enteritidis, *S*. Mbandaka, *S*. Javiana, *S*. Caracas, *S*. Ohio, *S*. Braenderup and *S*. Georgia in Trinidad [64] and *S*. Enteritidis in Barbados [65].

According to the Caribbean Public Health Agency (CARPHA) State of Public Health report [6], *S*. Kentucky, *S*. Montevideo, *S*. Javiana and *S*. subspecies enterica, which were found in the current study, were amongst the top 15 human *Salmonella* serotypes detected in the region as well as the most common *Salmonella* serotypes associated with human salmonellosis in Trinidad and Tobago between 2005–2012 and are therefore of public health significance. This is of importance since the most recent study in the country, prior to this project, was performed in 2006 [32] where only three serotypes (Kiambu, Kentucky and Mbandaka) were detected from the cottage poultry processors. In the current study, 10 serovars (*S*. Javiana, *S*. Kentucky, *S*. Manhattan, *S*. Aberdeen, *S*. Bloomsbury, *S*. Schwarzengrund, *S*. Molade, *S*. Montevideo, *S*. San Diego, *S*. Westhampton) were recovered. These serotypes may therefore have public health implications for consumers of improperly cooked chicken from the outlets studied.

Data from this study indicate the extent of contamination by *Salmonella* spp. in the selected outlets of cottage processors and supermarkets and the risk of salmonellosis occurring in consumers of contaminated, undercooked chicken meat sold at retail outlets in the country.

## Supporting information

S1 Appendix Sanitation score sheet for cottage poultry processor outlets. (DOCX)

S2 Appendix Questionnaire administered at pluck shops. (DOCX)
