# Supplementary material for: Prevalence and serotypes of Salmonella spp. on chickens sold at retail outlets in Trinidad
Source: PLoS One. 2018 Aug 23;13(8):e0202108. doi: 10.1371/journal.pone.0202108 (PMC6107152; doi:10.1371/journal.pone.0202108)
Supplement: S1 Appendix — (DOCX) [file pone.0202108.s001.docx]

**Appendix 1. Sanitation score sheet for cottage poultry processor outlets.**

| General sanitary condition at cottage poultry processor (Score sheet) | | | | | | |
| --- | --- | --- | --- | --- | --- | --- |
|  | Score | | | | | |
| Category | 1 (worst) | 2 | 3 | 4 | 5(best) | NA |
|  |  |  |  |  |  |  |
| 1. Handlers of chickens at outlet |  |  |  |  |  |  |
| 1.1 Cleanliness of clothes |  |  |  |  |  |  |
| Clean clothes with sleeves |  |  |  |  | X^a^ |  |
| Clean clothes without sleeve |  |  |  |  |  |  |
| Dirty clothes without sleeve |  |  |  |  |  |  |
| Dirty clothes with sleeves |  |  |  |  |  |  |
| Dirty clothes with dirty sleeves | X^b^ |  |  |  |  |  |
| 1.2 Wore aprons |  |  |  |  |  |  |
| Wore very clean aprons |  |  |  |  |  |  |
| Wore clean aprons |  |  |  |  |  |  |
| Did not wear aprons |  |  |  |  |  |  |
| Wore moderately dirty clothes |  |  |  |  |  |  |
| Wore very dirty aprons |  |  |  |  |  |  |
|  |  |  |  |  |  |  |
| 1.3 Had hair covered |  |  |  |  |  |  |
| Yes |  |  |  |  |  |  |
| No |  |  |  |  |  |  |
|  |  |  |  |  |  |  |
| 2. Cleanliness in cages or areas where |  |  |  |  |  |  |
| live birds are kept |  |  |  |  |  |  |
| Relatively clean and not crowded |  |  |  |  |  |  |
| Relatively clean and crowded |  |  |  |  |  |  |
| Relatively dirty- feces and  crowded |  |  |  |  |  |  |
| Relatively filthy and crowded |  |  |  |  |  |  |
| Very filthy and very crowded |  |  |  |  |  |  |
|  |  |  |  |  |  |  |
| 3. Sanitation in slaughter area |  |  |  |  |  |  |
| Kept very clean- little  blood/feathers/feces |  |  |  |  |  |  |
| Kept clean- some  blood/feathers/feces |  |  |  |  |  |  |
| Moderately kept clean-  blood/feathers/lot of feces |  |  |  |  |  |  |
| Poorly kept- blood/feathers/lot of  feces/few flies |  |  |  |  |  |  |
| Very poorly kept-  blood/feathers/feces/ many flies |  |  |  |  |  |  |
|  |  |  |  |  |  |  |
| 4. Sanitation in de-feathering |  |  |  |  |  |  |
| or 'plucking' area |  |  |  |  |  |  |
| Kept very clean- little  blood/feathers/feces |  |  |  |  |  |  |
| Kept clean- some  blood/feathers/feces |  |  |  |  |  |  |
| Moderately kept clean-  blood/feathers/lot of feces |  |  |  |  |  |  |
| Poorly kept- blood/feathers/lot of  feces/few flies |  |  |  |  |  |  |
| Very poorly kept-  blood/feathers/feces/many flies |  |  |  |  |  |  |
|  |  |  |  |  |  |  |
| 5. Sanitation in evisceration area |  |  |  |  |  |  |
| Kept very clean- little  blood/feathers/feces |  |  |  |  |  |  |
| Kept clean- some blood/feathers/feces |  |  |  |  |  |  |
| Moderately kept clean-  blood/feathers/lot of feces |  |  |  |  |  |  |
| Poorly kept- blood/feathers/lot of  feces/few flies |  |  |  |  |  |  |
| Very poorly kept-  blood/feathers/feces/many flies |  |  |  |  |  |  |
|  |  |  |  |  |  |  |
| 6. Sanitation in rinsing of carcases |  |  |  |  |  |  |
| Use of running water in sink |  |  |  |  |  |  |
| Use of 3 rinsing buckets/clean water |  |  |  |  |  |  |
| Use of 2 rinsing buckets/clean water |  |  |  |  |  |  |
| Use of 1 rinsing bucket/bloody  water/feathers |  |  |  |  |  |  |
| Do not rinse |  |  |  |  |  |  |
|  |  |  |  |  |  |  |
| 7. Sanitation in packaging and sale  areas |  |  |  |  |  |  |
| Kept very clean- no blood/feathers/  feces or flies |  |  |  |  |  |  |
| Kept clean- no blood/feathers or  feces but few flies |  |  |  |  |  |  |
| Kept moderately clean- some  blood/feathers/feces and flies |  |  |  |  |  |  |
| Poorly kept- blood/feathers/lots of  feces with few flies |  |  |  |  |  |  |
| Very poorly kept- some blood/lots of  feathers/lots of feces/many flies |  |  |  |  |  |  |

X^a^- Example of scoring of the overall cleanliness of handlers clothing: Clean clothes with sleeves- Score of 5 was recorded.

X^b^- Example of scoring of the overall cleanliness of handlers clothing: Dirty clothes with dirty sleeves- Score of 1 (worst) was recorded.
