# Supplementary material for: Prevalence and serotypes of Salmonella spp. on chickens sold at retail outlets in Trinidad
Source: PLoS One. 2018 Aug 23;13(8):e0202108. doi: 10.1371/journal.pone.0202108 (PMC6107152; doi:10.1371/journal.pone.0202108)
Supplement: S2 Appendix — (DOCX) [file pone.0202108.s002.docx]

**Appendix 2. Questionnaire administered at pluck shops.**

**THE UNIVERSITY OF THE WEST INDIES**

**FACULTY OF MEDICAL SCIENCES**

**SCHOOL OF VETERINARY MEDICINE**

**Prevalence and characteristics of *Salmonella* spp. isolated from retail outlets in Trinidad.**

**DATE: ________________ CODE: ______________**

**SECTION A- General information**

1. **Name of Pluck Shop:**

**___________________________________________________________**

1. **Address of Pluck Shop:**

**___________________________________________________________**

1. **Contact Person: _________________________________________**
2. **Telephone Number: _____________________________________**
3. **How many year(s) has the Pluck Shop been in operation? ______________________**

**SECTION B- Workers information**

1. **Number of workers (not including owner/owners):**

**Temporary (T) Number: _______________**

**Permanent (P) Number: _____________**

1. **Level of Training of workers:**

**Ministry of Health Number of workers: __________**

**Ministry of Agriculture Number** of workers**: ___________**

**Caribbean Poultry Association Number** of workers**: ___________**

**Other(specify): ____________________________ Number** of workers**: ___________**

1. **Length of time working in industry:**

**< 1 year……………Number of workers: ______**

**1-3 years…………...Number of workers: ______**

**3-5 years…………...Number of workers: ______**

**5-7 years…………...Number of workers: ______**

**>7 years…………...Number of workers: _______**

**Owner experience in the industry:  <1 year  1-3 years  3-5 years**

**5-7 years  >7 years**

**SECTION C- Operational Information**

1. **What is your average sale of broilers :**
2. **Mon- Fri (Or Weekdays): _________________**
3. **Sat-Sun: ________________**
4. **How many days is the pluck shop open per week? ______________________**
5. **What is the source of your live birds?**

**Arawak  Malabar Farms  Nutrimix**

**Master Mix Feed Mill  Warner Grain Mill (WGM)  Self-growing**

**Other (specify): _____________________**

1. **What method is used to rinse carcasses?**

**Sink  Drum/Tank  Other (Specify): ___________________**

1. **If Drum/Tank is used, how often is the rinse water changed?**

**every 20 carcasses or less processed**

**every 21- 40 carcasses processed**

**every 41- 75 carcasses processed**

**> 100 carcasses processed**

**once a day**

**never**

1. **Are there any quality control measures or practices in effect at the shop? (eg. How often do you perform general and complete cleaning of equipment/working areas)**

**Yes  No**

**If Yes, please describe the following:**

**General cleaning: _________________________________________________________**

**Thorough cleaning: _______________________________________________________**

**Do you use a chilled water bath after processing to cool carcasses before packaging?**

Yes  No

**If Yes to above, how long do you leave the carcass to chill in the water bath? ______mins**

**How often are pens where live birds are kept pre-slaughter cleaned? ________________________________________________________**

**What material is the flooring of pens made of? Dirt flooring Concrete flooring**

**Other measures: _______________________________________________________________________________________________________________________________________________________________________________________________________________________**

1. Do you sell carcasses from the counter top?

**Yes  No**

**If Yes, please state length of time before carcasses stored on counter tops are sold or stored in a chiller: ________mins**

1. Please list the complaints you received from your customers.

________________________________________________________________________________________________________________________________________________________________________________________________________________________

**Thank you for your cooperation!**
